# Epigenetic Regulation of Neuroinflammation in Parkinson’s Disease

**DOI:** 10.3390/ijms22094956

**Published:** 2021-05-07

**Authors:** Madiha Rasheed, Junhan Liang, Chaolei Wang, Yulin Deng, Zixuan Chen

**Affiliations:** School of Life Science, Beijing Institute of Technology, Beijing 100081, China; madiharasheed@bit.edu.cn (M.R.); 3220191185@bit.edu.cn (J.L.); 3220201326@bit.edu.cn (C.W.); deng@bit.edu.cn (Y.D.)

**Keywords:** Parkinson’s disease, neurodegeneration, neuroinflammation, epigenetics, astrocytes, microglia

## Abstract

Neuroinflammation is one of the most significant factors involved in the initiation and progression of Parkinson’s disease. PD is a neurodegenerative disorder with a motor disability linked with various complex and diversified risk factors. These factors trigger myriads of cellular and molecular processes, such as misfolding defective proteins, oxidative stress, mitochondrial dysfunction, and neurotoxic substances that induce selective neurodegeneration of dopamine neurons. This neuronal damage activates the neuronal immune system, including glial cells and inflammatory cytokines, to trigger neuroinflammation. The transition of acute to chronic neuroinflammation enhances the susceptibility of inflammation-induced dopaminergic neuron damage, forming a vicious cycle and prompting an individual to PD development. Epigenetic mechanisms recently have been at the forefront of the regulation of neuroinflammatory factors in PD, proposing a new dawn for breaking this vicious cycle. This review examined the core epigenetic mechanisms involved in the activation and phenotypic transformation of glial cells mediated neuroinflammation in PD. We found that epigenetic mechanisms do not work independently, despite being coordinated with each other to activate neuroinflammatory pathways. In this regard, we attempted to find the synergic correlation and contribution of these epigenetic modifications with various neuroinflammatory pathways to broaden the canvas of underlying pathological mechanisms involved in PD development. Moreover, this study highlighted the dual characteristics (neuroprotective/neurotoxic) of these epigenetic marks, which may counteract PD pathogenesis and make them potential candidates for devising future PD diagnosis and treatment.

## 1. Introduction

Parkinson’s disease (PD) is the second most common neurodegenerative disorder, after Alzheimer’s disease (AD), affecting the old age population [1]. It is characterized by the subclinical feature of cytoplasmic proteinaceous inclusions of Lewy bodies (LBs) in the substantia nigra (SN) of the PD brain and degeneration of dopaminergic neurons (DA). Extensive degeneration of dopaminergic neurons lowers dopamine levels in the brain, resulting in clinical manifestations such as postural instability, static tremor, bradykinesia, and ankylosis arthritis [2,3,4]. Following the dopaminergic loss, various non-dopaminergic neurons also degenerate and lead to several dopamine-resistant symptoms, including insomnia, olfactory dysfunction, autonomic dysfunction, pain, and sensory manifestations [5]. Though these symptoms are treated at primary stages with dopaminergic drugs (Levodopa), their effectiveness reduces with symptoms severity due to successive underlying neurodegeneration [2,6]. Hence, it makes PD treatment quite challenging and complicated.

For decades, the underlying pathophysiology of the PD has remained unclear. It is believed to be caused by both genetic mutations (*SNCA*, *LRRK2*, *PARKIN*, and *DJ-1*), termed as familial PD, and environmental factors (exogenous neurotoxins, age, diet, and lifestyle) in sporadic PD [7]. It is observed that these factors induce various molecular and cellular events, such as oxidative stress, α-synuclein oligomerization, mitochondrial dysfunction, higher iron concentration to trigger neurodegeneration and neuroinflammation. Nevertheless, the core molecular and cellular processes involved in the PD are divergent. However, all of these events participate over time in neuronal apoptosis by activating the microglia and astrocytes population in particular regions of the brain [5]. Any initial stimulus, either in the form of cellular stresses (genotoxic, osmotic, hypoxic, or oxidative stress) or environmental stresses (exogenous neurotoxins or stressful lifestyle), activates the neuronal immune responses, including microglia, astrocytes, inflammatory cytokines, etc. [8]. However, the continual activation of the neuronal immune system leads to a vicious cycle of neuroinflammation that causes chronic inflammatory environments that are likely to ensue neuronal dysfunction and ultimately the death of the endangered neuronal population. A significant number of PD studies, including animal and human models, have strongly linked neuroinflammation as environmental stressors that trigger progressive degeneration of DA neurons. Therefore, on-time delivery of anti-inflammatory therapy is required to provide neuroprotective effects in PD patients at risk of genetic and epigenetic dysfunction.

**Figure 1:**
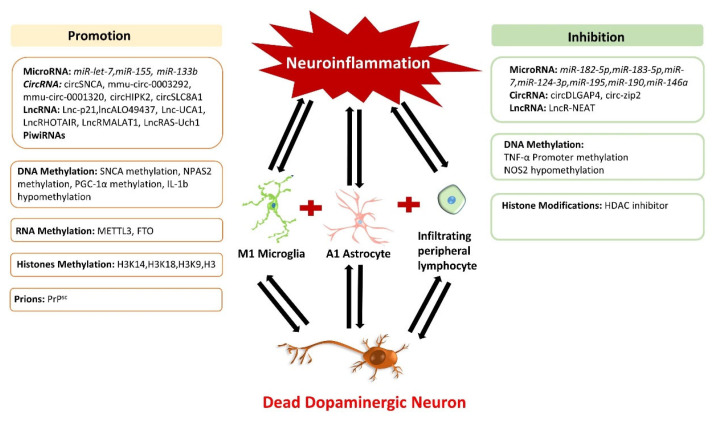
Impact of epigenetic mechanisms regulating neuroinflammation in Parkinson’s disease. The neuroinflammation cycle is activated on DA neuron damage, which triggers epigenetic modifications and disturbs the normal function of inflammatory responses. Epigenetic regulators are divided into two categories: neurotoxic and neuroprotective. Neurotoxic epigenetic regulators enhance inflammatory factors and ROS production, transform glial cells to an inflammatory phenotype, and promote dopaminergic neuron death. In contrast, neuroprotective epigenetic regulators display therapeutic characteristics and inhibit neuroinflammation by alleviating DA neuronal damage in Parkinson’s disease.

Epigenetic factors have been observed to play a dynamic role in regulating neuroinflammation in both forms of PD. These factors, including DNA/RNA methylation, histone modification, chromatin remodeling, non-coding RNAs (ncRNAs), etc., are sought to bridge environmental factors and genes by altering gene expression and chromosome function without changing the DNA sequence. Moreover, these epigenetic mechanisms regulate the immune cells and inflammatory factors and are believed to be part of the underlying mechanism countering neuroinflammation-induced DA damage in PD patients. It is worth mentioning that these epigenetic mechanisms act synergistically and do not regulate the neuroinflammation in PD independently. For instance, circRNAs and lncRNA sponge with miRNAs and regulate various target genes that activate microglia and astrocytes, thus trigger neuroinflammation in PD. Similarly, methylases cooperate with ncRNAs to activate various inflammatory pathways to decide the fate of glial cells in PD. Unfortunately, detailed studies of epigenetic mechanisms and their synergic interaction with neuroinflammatory pathways in PD have not been discussed until now. Therefore, in this review, we have discussed the impact of epigenetic mechanisms in regulating neuroinflammation in PD and identified their synergic interactions with various neuroinflammatory pathways. In addition, we have also highlighted various epigenetic modifications based on their neuroprotective and neurotoxic properties to identify their potential use in future PD management (Figure 1).

## 2. Crosstalk of Neuroinflammation in PD Progression

Neuroinflammation acts as a double-edged sword in the nervous system. Under normal circumstances, microglia and astrocytes protect against pathogenic attack or cellular stress. However, during PD progression, inflammatory cytokines activate glial cells and change their phenotype. This process triggers inflammatory mediators, reactive oxygen species (ROS), and nitric oxide synthase (NOS) production, leading to massive dopaminergic neuronal apoptosis and α-synuclein aggregation, thus intensifying PD development. Although the initial damage of DA neuron does not trigger neuroinflammation, the dead remains of DA neurons usually induce chemokines release that promotes the penetration of activated microglia to clear the dead remains of nerve fragments and thus results in neuroinflammation [9]. In 2016, Gerhard et al., performed PET scans on the brains of PD patients. They observed an increased level of neuroinflammation in the pons, basal ganglia, striatum, frontal, and temporal cortex areas along with the massive apoptosis of DA neurons [10]. Thus, it proposed that the apoptotic process of DA neurons and the development of neuroinflammation occurs simultaneously. Moreover, it is observed that the midbrain region enriched with DA neurons possesses the highest number of microglia [11]. Unlike neurons in the hippocampus or cortex, midbrain DA neurons exhibited more sensitivity for cytokines such as tumor necrosis factor (TNF) [12,13] and were directly associated with inflammation. In conclusion, the consistent degeneration of dopaminergic neurons in PD patients’ brains are the outcomes of chronic neuroinflammatory reactions.

### Molecular Insight of Neuroinflammation in Parkinson’s Disease

Neuroinflammation occurs on the activation and proliferation of microglia and astrocytes. Microglia are immune cells in the brain that participate in the inflammatory response in the central nervous system (CNS). Astrocytes are the most abundant type of glial cells in the CNS that support brain structure. When inflammatory factors such as TNFα and interleukins (IL-6, IL-1β, and IFN-γ) are recognized and received, microglia differentiate into the pro-inflammatory M1 phenotype. M1 phenotypic microglia secrete TNF-α, IL-6, IL-1β, ROS, and nitric oxide (NO) and reduce the secretion of neurotrophic factors to aggravate the damage of nerve cells [14,15]. In contrast, when stimulated by IL-4, IL-10, and other cytokines, microglia differentiate into the anti-inflammatory M2 phenotype. Microglia with M2 phenotype produces various anti-inflammatory compounds and has an immunosuppressive effect by antagonizing M1 microglia [15]. Microglia with M2 phenotype can also secrete IL-4, IL-13, TGF-β, and neurotrophic factors such as IGF-1 (insulin-like growth factor-1), displaying a neuroprotective role [14,15]. Similar to the microglia, astrocytes A1 phenotype is also programmed to secrete neurotoxic pro-inflammatory mediators after receiving inflammatory factors. However, while receiving anti-inflammatory factors, astrocytes with A2 phenotype produces neurotrophic factors and show neuroprotective role. Thus, the abnormal or over-activation of microglia and astrocytes M1/A1 phenotype and lower M2/A2 phenotype can be a possible mechanism that induces neuroinflammation and neurodegeneration in PD.

The underlying mechanism involved in chronic neuroinflammation is currently elusive and is thought to be affected by many factors. According to D Cherry’s review, the M1/M2 phenotype of microglia is mostly related to the transformation of acute neuroinflammation to a chronic state. It is observed that failure of the M1 to M2 phenotype of microglia reduces neurotropic factors (such as IGF1 or brain-derived neurotrophic factor) and leads to the production of inflammatory factors and ROS, which causes chronic neuroinflammation and results in neuronal apoptosis [16]. The M1/M2 phenotype transition of microglia is associated with cytokines and growth factors. For instance, when IL-6s are released, the JAK/STAT signaling pathway activates both *STAT1* and *STAT3* genes that triggers other neuroinflammation-related genes such as Jmjd3 and pro-inflammatory cytokines, thus promotes microglia-mediated neuroinflammation [17]. Contrarily, anti-inflammatory signal factors such as IL-10 and STAT3 would induce the expression of the SOCS1 gene and other anti-inflammatory genes to inhibit the pro-inflammatory response, mediated by TLR4 and IL-6 [18,19]. In addition, some regulatory drugs also change the phenotype of microglia. Such as LPS (lipopolysaccharide), TNF-α inducer stimulates the polarization of microglia into the M1 phenotype. Adding fasudil (Rho-kinase inhibitor) can reduce the activities of inflammatory cytokines IL-1β, IL-6, TNF-α, and NF-κB and trigger microglia towards the M2 phenotype [20]. Moreover, the NADPH inhibitor enzyme convert LPS induced M1 microglia into M2 [21]. Moreover, epigenetic modifications may affect the M1/M2 phenotype transition of microglia. For example, miR-155 drive microglia to M1 phenotype [22].

In PD, astrocytes induce the death of dopaminergic neurons by secreting the pro-inflammatory cytokines and other toxic molecules (NO, etc.) [23]. In contrast, there are other reports that astrocytes protect neurons by inhibiting neuroinflammation [24]. The paradoxical performance of astrocytes in PD neuroinflammation is due to its different polarization phenotypes. Excessive activation of the A1 phenotype and lack of A2 phenotype are important factors in developing neuroinflammation in PD. Cytokines and growth factors regulate the A1/A2 phenotype transition of astrocytes. For instance, the release of glycoprotein (gp130) leads to the A2 phenotype by astrocytes. Moreover, gp130 activated *STAT1* and *STAT3* and mediated the SHP2/Ras/ERK signal cascade to limit the neuroinflammation development. [25]. However, when IL17 was released, it bound to the heterologous transmembrane receptors, which led to the recruitment of NF-kB activator 1 (Act1) and the formation of signal complexes, thereby exhibiting the A1 phenotype and promoting the production of pro-inflammatory cytokines [26]. Except for cytokines, PD-related genes could also regulate the phenotype of astrocytes. For example, researchers found altered gene expression in astrocytes when exposed to α- synuclein and accelerated the inflammation process. They speculated that the *SNCA* gene might activate astrocytes in PD through IFN-c and TNF-α and regulate inflammation progression [27,28,29]. Similarly, in mouse models of PD, mutations in Parkin cause astrocyte dysfunction and aggravate neuronal death [30]. However, DJ-1 induces the A2 phenotype of astrocytes through the STAT1 pathway and protected neurons by inhibiting brain inflammation in PD patients [28,31,32].

Although the source and structure of microglia and astrocytes are quite different, they work mutually in any stimulus (brain injury). The synergy between them in neurodegenerative diseases has become a hot point in recent years. According to Ilia D. Vainchtein’s review, both microglia and astrocytes have specific receptors for norepinephrine, purines, and bacterial metabolites in the blood, which seem to induce synchronous reactions between them. Transforming growth factors (TGF-β) released by astrocytes could let neurons release certain components which act on microglia while astrocytes and microglia interact directly with molecules as IL-1α, TNF-α, and IL-33. For example, IL-1α released by astrocytes changed the permeability of the blood–brain barrier, thereby causing the activation of microglia [33]. The activated microglia secreted Il-1α, TNF-α, and C1q both in vivo and in vitro, which induced A1 astrocytes to function [34]. In PD, the two coordinated regulation mechanisms jointly regulate the progression of neuroinflammation. Research by Kaoru Saijo et al., showed that *Nurr1* (a monocyte receptor) inhibited the expression of pro-inflammatory neurotoxic mediators in microglia and astrocytes and reduced the death of DA neurons. Decreased expression of *Nurr1* made astrocytes more susceptible to the influence of microglia and promoted inflammation [35]. Hayate Javed et al., found that glial maturation factor (GMF) was a pro-inflammatory factor, which may be a co-receptor of microglia and astrocytes. However, inhibiting GMF reduced the activation of NLRP3 (NLR family pyrin domain containing 3) inflammasomes and regulated the cytotoxic function of microglia and astrocytes, thus prevented PD development [36]. Studies by Bortolanza et al., also showed that 7-nitroindazole (a NOS inhibitor) could reduce the iNOS expression in PD rat models to downregulate neuroinflammation by inhibiting the activation of astrocytes and microglia [37]. Thus, more research in this domain may provide valuable insight into the deep understanding of the underlying mechanism of glial cell coordination and communication that may help researchers identify the novel targets for PD therapy.

## 3. Epigenetics and Neuroinflammation in PD

Epigenetic modifications possess a dual function in the regulation of the nervous system. Their neurotoxic and neuroprotective properties make them ideal candidates for disease management. Ample studies have shown that neuroinflammation in PD is regulated by various epigenetic modifications such as non-coding RNA, DNA/RNA methylation, and histone acetylation. They regulate neuroinflammation by governing immune cells, including macrophages, T lymphocytes, microglia, and astrocytes. For instance, histone 3 lysine 27 (H3K27) methylation enhances the inflammatory phenotype of macrophages and microglia, which is known as the M1 response. In contrast, H3K27 histone demethylase jumonji-domain Protein 3 (Jmjd3) upregulates the anti-inflammatory M2 phenotype of microglia [38]. Thus, a detailed study of these epigenetic mechanisms may unravel the hidden mystery of neuroinflammation involved in PD progression.

### 3.1. Non-Coding RNA and Neuroinflammation

#### 3.1.1. MicroRNAs

MicroRNAs belongs to the class of small non-coding RNA (sncRNA) with a length of 17–22 nt. They inhibit gene expression level by binding to the 3’untranslated region (3’UTR) of mRNA, resulting in mRNA degradation, adenylation, or translational inhibition [39]. Over the past few years, various miRNAs target PD-related genes that induce neuroinflammation and accelerate the neurodegeneration of dopaminergic neurons (Table 1). For instance, downregulation of miR-133b in the mid-brain tissues of Parkinson’s patients has resulted in the malfunctioned dopaminergic neurons and induces neuroinflammation in PD [40]. Another study reported that dysregulated Let-7 had promoted neuroinflammation through the activation of microglia and macrophages by acting as DAMP of TLR7 (Toll-like receptors 7) that further induced microglia to secrete inflammatory factors [41,42,43]. Contrarily, Let-7 also promoted the M2 phenotype in microglia by targeting the C/EBP-δ transcription factor and inhibiting neuroinflammation-induced apoptosis [44,45]. Similarly, *SOCS1* and *SOCS3* genes involved in regulating the inflammatory signal transduction of microglia were found downregulated by miR-155 and enhanced neuroinflammation in PD [46].

Interestingly, various microRNAs possess a therapeutic potential and provide neuroprotection against neuroinflammation by targeting PD-associated genes (Table 1). In 2018, Roser et al., reported the neuroprotective role of miR-182-5p and miR-183-5p, which downregulated *GDNF* (glial cell-derived neurotrophic factor) and protected DA neurons against neuronal damage in the PD model [49]. A cytoplasmic protein NLRP3 belongs to an inflammatory signaling complex, inflammasome, plays a significant role in inflammation-mediated pyrophosphorylation neurodegeneration of DA in substantia nigra par compacta (SNpc) in PD [55,56,57]. A study performed on the striatum of the PD mouse model showed that miR-7 lowers the expression of the *NLRP3* gene and *SNCA*, which resulted in reduced neuronal damage and improved microglia function in PD [47,48]. Another study on the PD model showed that inhibition of *STAT3* (transcription factor regulating monoamine oxidase A) and *MAPK* (mitogen-activated protein kinase) pathway reduced neuroinflammation and behavioral changes caused by microglia [58]. miR-124-3p was observed to exert its neuroprotective role in DA neurons by downregulating *STAT3* gene expression in the PD cell model induced by MPP [50]. In another study, miR-124 targeted the *MEKK3* in the NF-Kb pathway and the nuclear factor kappa light chain enhancer that reduced inflammatory cytokine levels in the PD mouse model [51].

Other than targeting inflammation-related genes in PD, miRNAs are also involved in regulating microglia and astrocytes. Recently, PD pathogenesis was investigated on LPS induced in vitro model for microglia activation. This study showed that upregulated miR-195 inhibited the release of pro-inflammatory cytokines such as inducible nitric oxide synthase, IL-6, and TNF-a and increased the release of anti-inflammatory cytokines IL-4 and IL-10. Moreover, miR-195 was observed to downregulate *ROCK1* gene expression in the PD model, which resulted in lowered microglia activation and reduced neuroinflammation [52]. Higher expression of miR-190 in the PD model inhibited pro-inflammatory mediators such as iNOS, IL-6, TNF-α, and TGF-β1 and enhanced the release of anti-inflammatory mediators, such as IL-10. Thus, it postulates that miR-190 negatively regulates the expression of the *Nlrp3* gene to inhibit the activation and inflammatory response of microglia, which abridged neuronal damage in SNpc [53]. 

Another study on PD mouse models treated with LPS and IFN-γ showed differential expression of miRNAs in cortical astrocytes. For instance, miR-146a, miR-146b, and miR-155 were found upregulated, whereas miR-351, miR-455, and miR-149 were downregulated. These subsets of differential miRNAs regulated TNF-α signaling pathway genes and activated astrocyte immune response in the PD [54,59]. Further investigation by Iyer et al., in 2012, demonstrated that upregulated miR-146a displayed neuroprotection by reducing the expression of IL-6 and COX-2 and inhibited the inflammatory response mediated by astrocytes [54]. Therefore, through the previous literature, it was observed that most of the miRNAs involved in neuroinflammation are interlinked and induce subsequent molecular changes along with neuroinflammation in the PD. Thus, it is difficult to predict the exact mechanism of miRNAs involved in PD progression at this stage and requires more researcher’s attention to gain insight into the underlying PD-associated molecular mechanism. Nevertheless, neurotoxic and neuroprotective properties of these miRNAs propose their potential as candidate biomarkers for specific diagnostics and therapeutics of future PD patients.

#### 3.1.2. CircRNA

CircRNAs are naturally occurring endogenous ncRNAs, widely distributed throughout the body. They are single-stranded RNA molecules up to 100 nucleotides in length and are covalently linked with 3′ to 5′ ends to form a loop-like structure [60]. They are highly expressed in the brain and regulate various gene expressions by acting as a sponge with miRNAs. Due to the higher percentage in the brain, they regulate various neuronal processes, where their deregulated expression footmarks several neurological disorders. During PD progression, various circRNAs sponge with miRNA to induce neuroinflammation along with other sub-molecular manifestations (Table 2).

The neuroprotective microRNA miR-7 was found downregulated due to the sponging effect of ciRS-7 and circSNCA in PD models. Downregulated miR-7 upregulated the *SNCA* gene expression that resulted in α-synuclein aggregation accompanied by higher oxidative levels, mitochondrial dysfunction, neuroinflammation, and neuronal death [70]. Contrarily, the downregulation of circSNCA under pramipexole (PPX) treatment proposed its therapeutic effect, reduced PD cell apoptosis, and improved autophagy [61]. Recently, transcriptomic profiling of PD brains of a mouse model showed that mmu-circRNA-0003292 sponges with miRNA-132 to downregulate the expression of the *NR4A2* gene [62]. Lower expression of *NR4A2* caused impaired development and differentiation of midbrain neurons that promoted DA neurodegeneration and neuroinflammation, hallmark parkinsonism [62,71]. Another investigation on the PD model showed that circSLC8A1 sponged with miR-128 and downregulated *SIRT1* and *BMI1* transcripts. This circSLC8A1/miR-128 sponged influences protein homeostasis and chronic neuroinflammation mitochondrial dysfunction and promotes neurodegeneration to induce PD progression [66,67,68].

Since the past few years, circRNAs have gained great attention due to their neuroprotective effects against PD pathogenesis. Recently, Zhong Feng et al., showed that circDLGAP4 exerts its neuroprotective effect through regulating the miR-134-5p/CREB pathway in human and mouse PD models [63]. Moreover, circDLGAP4 sponged with miR-134-5p to regulate the activation of CREB pathway and CREB-associated genes, such as *BDNF*, *Bcl-2*, and *PGC-1α*, which promoted neuron viability by reducing mitochondrial dysfunction, neuroinflammation, and enhanced autophagy [72,73,74]. In another study, mmu_circRNA_0001320 sponged with miRNA-124 and inhibited neuroinflammation in PD by regulating the MEKK3/NF-κB signaling pathway [62,69]. Thus, it speculates that the mmu_circRNA_0001320/miRNA-124/MEKK3 axis plays an integral part in regulating the levels of the inflammatory factors in PD neuroinflammation. Knockdown of circHIPK2 in the PD model presented therapeutic properties against neuroinflammation. It caused lower expression of MIR125-2HG and SIGMAR1 gene expression and inhibited astrocyte activation through the modulation of autophagy and reduced endoplasmic reticulum stress levels [64]. Circzip-2 has been reported to protect the dopaminergic neurons by targeting miR-60 that downregulated the zip-2 expression. This circzip-2/miR-60 axis provided resistance against oxidative stress and reduced neuroinflammation in the PD model [65]. Therefore, due to their precise involvement and stability in PD development, it is proposed that circRNAs can serve as a potential tool for PD management strategies.

#### 3.1.3. LncRNA

Long non-coding RNA (lncRNAs) is a RNA transcript with a length greater than 200 nucleotides [75]. LncRNAs are highly expressed in the central nervous system and regulate several neurobiological processes such as neural plasticity, neurogenesis, brain development, etc., where any dysregulation results in various neurodegenerative diseases [75]. Converging evidence has reported that various lncRNAs are involved in PD progression, as summarized in Table 3. In 2017, Ni Y et al., reported differential expression of 87 lncRNAs in the substantia nigra of PD patients [76]. Whereas 13 lncRNAs were found differentially expressed in the peripheral blood leukocytes of PD patients [77], postulating that lncRNAs actively participate in PD pathogenesis.

LncRNAs display an integral role in neuroinflammation development in PD. For instance, lncRNA UCA-1 (urothelial carcinoma-associated-1) is reported to induce apoptosis and neuroinflammation in the PD model through targeting P13K/Akt signaling pathways. However, its knockdown has reduced oxidative stress and inflammatory responses, which lowered the degeneration of dopaminergic neurons [78]. Another investigation showed that LncRNA-p21 (long non-coding RNA-p21) binds with miR-625 to induce cytotoxicity and neuronal apoptosis in the PD cell model. In contrast, silencing of LncRNA-p21 has reduced cytotoxicity along with TNF-α, IL-1β, and IL-6 levels, which increased the SOD activity and reversed neuronal damage and neuroinflammation [83]. lncRNA HOTAIR has been found to target the miR-126-5p and RAB3IP in a ceRNA- dependent manner and enhanced PD progression [79]. Moreover, lncRNA HOTAIR and lncRNA-MALAT1 bind with miR-205 and downregulated *LRRK2* gene expression to induced oxidative stress, neuronal apoptosis, and neuroinflammation [80,86] Contrarily, downregulation of these lncRNAs nullified the α-synuclein aggregation and the apoptosis of dopaminergic neurons. Further studies showed that lower expression of lncRNA ASUchl1 (antisense to the mouse Ubiquitin carboxy-terminal hydrolase 1) in the PD model had downregulated *Nurr1* gene expression, which also contributed to neuronal damage and associated neuroinflammation [82]. Though a wide range of lncRNAs is found dysregulated in PD, but still not enough to understand the exact mechanism of lncRNAs in PD development. Therefore, detailed investigations are required to figure out the exact mechanism involved in PD progression. 

In addition to the neurotoxic role of lncRNAs in PD, several LncRNAs displayed therapeutic significance by protecting neurons against neuroinflammation and oxidative stress in PD models. lncRNA NEAT (nuclear-enriched assembly transcript-1) has been reported to exert a neuroprotective role by upregulating the *PINK1* gene expression, which inhibited the *PINK1* protein deterioration in PD models and reduced neuronal injury and neuroinflammation [85]. Recent research by Zhang et al., showed that silencing of lncRNA AL049437 had reduced TNF-α, iL-6, and ROS production in MPP^+^ induced PD model, which significantly reduced the neuroinflammation and oxidative stress [84]. Though a limited number of lncRNAs are reported with neuroprotective properties, it still broadens the vision for their useful application in PD therapeutics.

#### 3.1.4. Piwi Interacting RNAs

Piwi-interacting RNAs (piRNAs) are small non-coding RNAs (26 to 31 nt), referred to as genomic guardians. They are involved in protecting the genome by facilitating the transcriptional and post-transcriptional silencing of transposable elements through DNA methylation and RNA interference. It is recently reported that piRNAs regulate various brain functions and are involved in various neurological diseases, including PD [87,88].

An investigation on PD patients reported lowered expression of *SINEs* (short interspersed nuclear element) and *LINEs* (Long interspersed nuclear element), which caused significant alterations in *PGC-1α* and CREB-pathways, resulted in the mitochondrial dysfunction and associated neuroinflammation [89]. Further study on long-term memory in the Aplysia CNS showed that piRNA plays a key role in regulating the methylation of *CREB2* gene promoter region [90]. Thus proposes that piRNAs might lower *CREB* gene expression that progresses neuroinflammation in PD patients [91]. So far, limited studies have been reported in this domain; therefore, we require extensive research to elucidate their role in the pathogenesis of PD.

### 3.2. Methylation Regulation and Neuroinflammation

#### 3.2.1. DNA Methylation

DNA methylation is an epigenetic modification that involves the covalent transfer of a methyl group to the C-5 position of cytosine by DNA methyltransferase (DNMT) to induce chromatin conformation and inhibits the transcription mechanism, which results in abnormal gene expression [92]. DNA methylation is important for normal body development. It plays a vital role in various processes such as genomic imprinting, inactivation of X-chromosome, and downregulation of repetitive element transcription and transposition, where any deregulation results in various diseases, including neurodegenerative disease. A significant number of disturbed methylation patterns have been observed in various genes that trigger neuroinflammation and PD development, summarized in Table 4. Accumulating evidence showed that lower methylation levels in *SNCA* mRNA of PD patients resulted in the higher translation of *SNCA* mRNA and α-synuclein aggregation [93,94,95]. Similarly, another investigation by Masliah et al., showed that nuclear DNMT1 levels were reduced in PD brain samples and *SNCA* transgenic mouse models, leading to insufficient DNA methylation in the CpG island upstream of the *SNCA*, *SEPW1*, and *PRKAR2A* genes [96]. Interestingly, the methylation pattern of the *SNCA* gene in the brain was found similar to the blood of PD patients [97]. Hence DNA methylation patterns from peripheral blood of PD patients can serve as substitute biomarkers for PD progression [98].

TET enzyme encoded by the TET gene family is reported to play a prominent role in DNA demethylation. A study performed by Li Shu et al., on 1657 PD patients and 1394 control subjects showed that the TET1 gene might influence PD risk by regulating the 5hmC levels and subsequent gene expression [104]. Likewise, the Clock gene that controls circadian rhythm presented significant variations in its expression in PD patients and animal models [99,105,106]. Investigation of the seven clock genes (*PER1*, *PER2*, *CRY1*, *CRY2*, *Clock*, *NPAS2*, and *BMAL1*) revealed that PD patients’ leucocytes showed reduced *NPAS2* promoter methylation, whereas the rest of the clock genes’ promoters found unmethylated [99]. Isil Ezgi Eryilmaza et al., in 2017, observed that variations in the methylation patterns increased the transcription regulation of *PARK2*, which resulted in mitochondrial dysfunction, dopaminergic neuron apoptosis, and neuroinflammation in PD [107]. Overall, these studies indicate that DNA methylation and demethylation play a significant role in the pathological process of PD.

PGC-1α is a transcriptional co-regulator, which is found downregulated during PD pathogenesis [107]. Analysis of human PD brain samples showed increased methylation at the *PGC-1α* promoter region. In evidence of this study, ICV administration of palmitic acid (induces epigenetic modification in neurons, microglia, and astrocytes) to α-synuclein transgenic mice resulted in hypermethylation of the *PGC-1α* promoter in the substantial nigra (SN), leading to the lower expression of *PGC-1α* gene and mitochondrial content along with higher expression of inflammation-related genes [101]. However, increased expression of the *PGC-1α* gene in PD models has presented the neuroprotective effects in PD models by increasing the expression of mitochondrial respiratory chain nuclear coding subunits and hence, prevented the loss of dopaminergic neurons [108]. In another study of active multiple sclerosis MS, Philip G Nijland et al., 2014 found that overexpression of *PGC-1α* in astrocytes could significantly reduce pro-inflammatory IL-6 and production and secretion of chemokine (CC motif) ligand 2. This led to reduced ROS production and inhibited oxidative damage and inflammation, thereby lowering the neurodegeneration [109]. Thus, we speculate that increased methylation of *PGC-1α* in PD may inhibit the neuroprotective effect of astrocytes and transform them to the A2 phenotype to promote neuroinflammation development.

During PD pathogenesis, the inflammatory factors induce neuroinflammation by changing the phenotype of microglia and astrocytes. Excessive activation of microglia led to the upregulation of TNF-α, il-1, il-6, il-12, and other pro-inflammatory molecules, along with mitochondrial ROS production and loss of mitochondrial membrane potential, which leads to neuronal damage [110]. Thus, TNF-α activates microglia to promote the transformation of their inflammatory phenotype, while the inflammatory response of microglia induces more production of TNF-α to form a vicious circle. Increasing evidence has proposed that higher TNF expressions malfunctioned the glutamate receptors that disturb the transportation of specific ions across nerve cells and lead to neurodegenerative diseases, such as AD and PD [111]. Various studies show that PD patients exhibited higher levels of TNF-α in their cerebrospinal fluid and TNF-α receptor 1 (TNFR 1, p55TNFR) in SNpc [112,113,114]. However, TNF-α knockdown inhibited microglia activation and reduced the neurotoxicity of MPTP in DA [114]. DNA methylation is significantly involved in regulating the expression of inflammatory factors. In 2008, Heike C. Pieper et al., reported that PD patient’s SNpc cells possess a lesser degree of DNA methylation in the TNF-α promoter than the DNA of other brain parts. Additionally, they observed that specific methylation in the CpGs dinucleotides of the TNF-α promoter activity lowered the binding of specific transcription factors (AP-2 and Sp1) that downregulated the TNF-α promoter activity which increased the sensitivity of dopaminergic neurons to TNF-α-mediated inflammation [102]. In summary, DNA methylation may regulate the phenotype of microglia and inflammatory response mediated by inhibition of TNF-α expression.

iNOS is a pro-inflammatory factor encoded by the *NOS2* gene and is highly expressed in the SNpc of PD patients. *NOS2* gene is mostly regulated at the transcriptional level or partly by the methylation of CpG dinucleotides [115]. Hypermethylation of the CpG site in the 5’promoter region of the *NOS2* gene reduces the iNOS activity [115,116]. In contrast, hypomethylation of *NOS2* may increase the iNOS activity. A study on PD patients exposed to welding fumes (mainly containing Mn) reported insufficient *NOS2* methylation [103], which resulted in higher iNOS activity. In contrast, inhibiting iNOS reduced the neuronal stress and downregulated the activation of microglia caused by signal molecules (such as MMPs), thus prevented neuronal damage [117,118]. Collectively, it can be deduced that the methylation level of *NOS2* affects the iNOS activity and regulates the activation of microglia and the neuroinflammation caused by it.

Interleukin 1 (IL-1) has been implicated in various neurological conditions due to its significant role in the central nervous system and neurodegeneration. Increased expression of IL-1β has been observed in the cerebrospinal fluid and striatal regions of patients with various neurological diseases, including PD, inducing neuronal death and damage [119,120,121]. Various PD studies reported that IL-1β induced neuroinflammation by disabling microglia. Hypomethylation of the IL-1β promoter region has been observed to convert microglia to the M1 phenotype, triggering neuronal damage and neuroinflammation [79,100]. Thus, methylation of IL-1β may be one of the influencing factors of PD and other diseases closely related to age.

#### 3.2.2. RNA Methylation

RNA methylation (m6A) is the most prevalent post-transcriptional modification of RNA, which involves binding the methyl group to the N6 site of adenine (RNA base). It is widely distributed in mRNA and long coding RNA (lncRNA). Most of the m6A modifications occur in exons, and it remains in the mature mRNA after splicing and influences the translation of m6A-containing mRNA. Methyltransferases and demethylases catalyze m6A modifications. Common methyltransferases include METTL3, METTL14, RBM15/B, etc., whereas demethylases include FTO (obesity-related protein) and ALKBH5 (alkylated DNA repair protein alkB homolog 5) [122,123,124,125]. Recently it has been observed that protein families containing the YTH domain may interact with m6A demethylases and contribute to the translation, degradation, and splicing of RNA [126]. Substantial evidence suggests that m6A modifications play a crucial role in numerous mechanisms, such as splicing, transportation, location, and stability of mRNAs, impacting various biological processes, including stem-cell differentiation, somatic-cell reprogramming, biological rhythms, etc. [127]. However, the actual mechanism of m6A modifications remains elusive and requires more investigations.

In the adult brain, m6A modifications are highly expressed. Substantial evidence has shown that dysregulation of m6A modifications is associated with many neuronal processes, such as dopaminergic signaling, neurogenesis [128], learning, and memory [129,130,131], proposing the close association of m6A modifications and brain activity. Recently, a study on the mouse model showed that dysregulated m6A modifications induced by circSTAG1 affected the stability of FAAH (an indispensable membrane enzyme that can degrade fatty acid amide family) mRNA, which caused astrocyte dysfunction and depressive behaviors [132]. Transcriptome-wide profiling of m6A modifications of mice with depressive behavior demonstrated a high degree of m6A modifications in the prefrontal cortex with lowered levels of FTO (nucleic acid demethylase), which resulted in the degradation of subsets of proteins involved in neuronal plasticity and led to the impaired memory-related processes [129,130,131].

More recently, m6A modifications have gained wide attention due to the significance of epitranscriptomic regulation in PD progression. A study performed on PD mouse and cell models showed that a high level of FTO reduced the m6A modifications in dopaminergic cells, resulted in the overexpression of NMDA receptor 1 (N-methyl-D-aspartate), oxidative stress, and Ca^2+^ influx, leading to neuronal damage and neuroinflammation [133]. Another investigation on m6A modifications in Chinese Han people with sporadic PD showed common and rare variations in m6A regulating genes (METTL3, METTL14, WTAP, FTO, ALKBH5, YTHDF1, YTHDF2, YTHDF3, HNRNPC, and ELAVL1). Gene-wise association analysis shows that no significant association has been found between these genes, and it is proposed that ethnic factors might restrict this association [134].

However, Methyltransferase-like 3 (METTL3), an RNA methyltransferase, is also involved in regulating various immune and inflammation processes. For instance, in an investigation on osteoarthritis, increased levels of IL-1β have been observed to enhance the expression of METTL3 that induced the significant m6A modifications. In contrast, METTL3 knockdown has reduced IL-1β-induced inflammatory cytokine levels and activation of NF-κB signaling, thus reduced neuronal damage [135,136]. Due to very limited research on m6A modifications in PD so far, the impact of METTL3 on neuroinflammation has not been studied for PD-associated m6A modifications. Though m6A modifications are widely dysregulated in PD, still this domain is barely touched and requires more investigation to understand the underlying mechanism in PD development.

### 3.3. Histone Modification

Histones are the basic structural proteins of chromosomes divided into H1, H3, H2A, H2B, and H4. They are involved in post-transcriptional modifications, including acetylation, phosphorylation, methylation, ubiquitination, sumoylation, ADP-ribosylation, etc., where variations in histones may lead to the dysfunction of the DNA transcription machinery through abnormal condensation of chromatin [137] and lead to various disorders. Various PD studies have shown a strong association between histone modification and neuroinflammation, implicating its role in PD progression.

Accumulating studies have shown a significantly higher level of histone modifications in PD models [138]. Increased histone acetylation is attributed to the imbalance between histone deacetylase (HDAC) and histone acetyltransferase (HAT). During PD development, downregulation of histone deacetylase 1 (HDAC1) induces an abnormal neuronal cell cycle, leading to DNA damage and neuronal apoptosis [139]. In 2016, Sugeno reported that α-synuclein directly bound to histones, which upregulated histone acetyltransferase and reduced the level of acetylated histone H3 in the PD cell model [140]. This interaction resulted in reduced sirtuin activity, abnormal α-synuclein aggregation, mitochondrial dysfunction, and oxidative stress, resulting in DA damage and neuroinflammation [141]. Further studies have shown that PD patients possessed increased acetylation of histone H3 in the brain samples due to increased acetylation of histone H3 lysine 14 (H3K14) and histone H3 lysine 18 (H3K18) and decreased deacetylation of histone H3 lysine 9 (H3K9). This higher level of H3 acetylation followed by the downregulation of histone deacetylase led to an over-acetylation of core histones, which inhibited the gene transcription in the primary motor cortex and accelerated the degeneration of dopaminergic neurons [142,143].

In an eleven-year follow-up of four million Norwegians, Shuchi Mittal et al., found that the β2AR agonist, salbutamol (a brain-permeable asthma drug), can effectively reduce the incidence of PD. Further studies have shown that β2AR ligand regulates the *SNCA*’s transcription by affecting the acetylation of histone 3 lysine 27 of *SNCA* promoter and enhancer [144]. Moreover, it is observed that the activation of microglia in PD is significantly related to the overexpressed histone acetylation. AGK2 is an inhibitor that targets the SIRT-2 site on HDAC and is reported to protect dopaminergic neurons and reduced microglial activation [145]. Similarly, nicotinamide (an amide converted from vitamin B3) also presented a neuroprotective effect in PD animal models [146]. Recently a study on the PD rat model showed that nicotinamide induced hyperacetylation of histones and increased the expression levels of various neurotrophic and anti-apoptotic factors in the brain, thereby presenting therapeutic effects against PD progression. However, nicotinamide overdose caused neurotoxicity and led to dopaminergic neuronal damage, suggesting an appropriate dose range is needed for effective therapeutic results [147]. Studies by Xuefei Wu et al., showed that HDAC inhibitors, including sodium butyrate (SB) and trichostatin A (TSA), upregulated the expression of *GDNF* and *BDNF* in astrocytes and inhibited the HDAC to protect DA neurons [148]. In rodent models of central nervous system inflammatory diseases, *BDNF* can prevent NF-κB-mediated neuroinflammation and apoptosis [149]. In addition, HDAC inhibitor TSA can also alleviate the MPP^+^-induced astroglial glutamate uptake disorder, which may be a new mechanism for HDAC inhibitors to promote neuroprotection [150].

### 3.4. Prions

Prions are lethal and pathogenic proteinaceous agents involved in various neurological disorders, including PD [151,152]. They possess epigenetic inheritance and can transmit genetic information to both mitotic and meiotic cell divisions without altering the genomic sequence [153]. Recently, the “prion transmission” of α-syn has been extensively studied in PD. It is observed that α-syn can spread throughout the body during the pathogenic process and promote microglial activation and neuroinflammation in PD. These activated microglia further promote the aggregation of α-syn and may provoke neuroinflammation, forming a vicious cycle of neurodegeneration of DA neurons in PD. Therefore, it affirms that neuroinflammation induces α-syn prion-like behavior to feed-forward neuronal damage. Moreover, it is also proposed that the gastrointestinal tract and olfactory epithelium are directly associated with neuroinflammation. Disturbed gastrointestinal tracts are the initial sites of α-syn accumulation presumed to be transmitted to the nervous system through “prion-like behavior” and tune progressive neuroinflammation [153]. Unfortunately, the underlying mechanism of α-syn accumulation in the intestinal track has not been elucidated until now, hence makes a loophole in the hidden mystery of PD origin. However, based on these studies, it can be hypothesized that dietary patterns and gut microbiota might play an active part in α-syn accumulation in the intestine and triggers PD onset through prion-like behavior. Further studies are required to unravel the exact pathogenic role of α-syn prions and their association with intestinal health and PD progression.

## 4. Synergistic Epigenetic Regulation and PD

**Figure 2:**
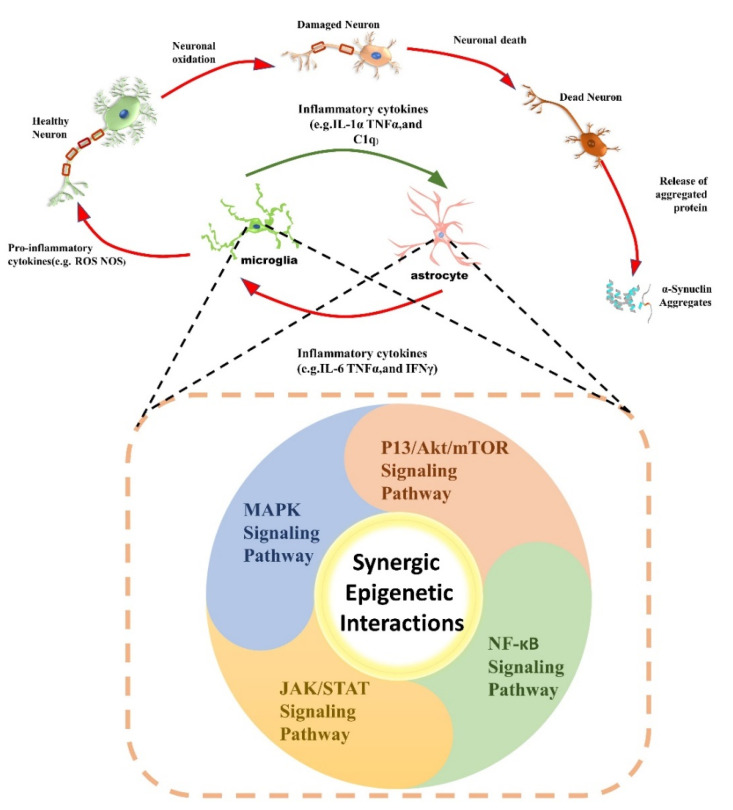
An overview of synergic epigenetic interactions with neuroinflammatory pathways in PD development. Various epigenetic mechanisms synergistically interact with each other and with inflammatory pathways (MAPK signaling pathway, P13K/Akt/mTOR signaling pathway, JAK/STAT signaling pathway, and NF-κB signaling pathway) to feedforward neuroinflammation in Parkinson’s disease. These neuroinflammatory pathways activate microglia and astrocytes and release inflammatory cytokines. Overactivation of microglia and astrocytes triggers the release of pro-inflammatory cytokines, which further damages normal dopamine neurons, leading to neuronal apoptosis and α-synuclein aggregation. Altogether, these synergic interactions form a vicious cycle that further exaggerates neuroinflammation to hallmark parkinsonism.

As discussed above, these epigenetic modifications play an integral role in developing neuroinflammation in Parkinson’s disease, but the exact underlying mechanism is still unclear and requires more investigations. However, through a literature survey, we attempted to find the correlation and contribution of these epigenetic modifications with various pathways involved in the progression of neuroinflammation, as shown in Figure 2. These synergic epigenetic interactions in neuroinflammatory pathways may provide a better understanding towards the biological mechanisms involved in neuroinflammation in PD.

### 4.1. MAPK Pathway in Neuroinflammation

MAPK signaling pathways play a dynamic role in key cellular functions, including cell division, differentiation, metabolism and motility, stress response, survival, or cellular apoptosis [154]. Dysfunctional MAPK signaling pathways contribute a significant role in the pathogenesis of various human ailments ranging from cancer to neurodegenerative diseases. During PD progression, stressful conditions, either environmental or cellular stresses or inflammatory cytokines (TNF-α and IL-1B), activate the microglial cells, which trigger the MAPK (mitogen-activated protein kinase) family, including p38MAPK and SAPK/JNK [155,156,157]. Moreover, p38 MAPK induces various inflammatory processes such as synthesis of cytokines, pro-inflammatory mediators, and iNOS and COX-2 [86,158]. In contrast, SAPK (stress-activated protein kinases)/JNK (Jun amino-terminal kinases) regulates the expression of inflammatory mediators such as COX-2 and iNOS and contributes to neuroinflammation [159].

To date, various genetic mutations and epigenetic mechanisms have been associated with the onset of PD. Accumulating studies have shown that abnormal expression of PD-associated genes is linked with neuroinflammation due to dysfunctional MAPK signaling pathways [160]. Abnormal expression of the *SNCA* gene is one of the underlying pathological causes of PD that leads to abnormal α-synuclein misfolding to form Lewy bodies in the SNcps of PD and impart neuronal toxicity [161,162,163]. Moreover, α-synuclein triggers p38, ERK, and JNK pathways in human microglial cells, leading to the production of IL-1β and TNF-α and subsequent promotion of neuroinflammation [164]. Similarly, α-Synuclein also regulates the expression level of IL-6 and intercellular adhesion molecule-1 (ICAM-1) in human astrocytes and accelerates chronic inflammation [165].

**Figure 3:**
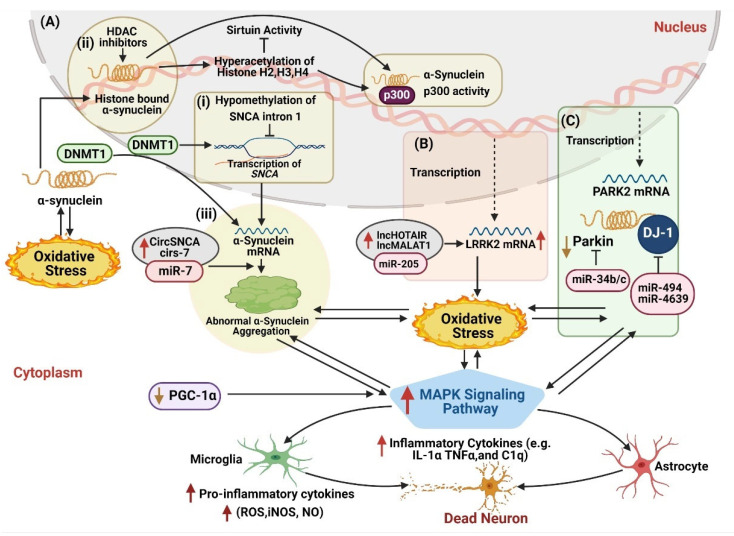
The landscape of synergic epigenetic interactions with MAPK signaling pathway in PD development. (**A**) MAPK signaling pathway is activated due to abnormal accumulation of α-synuclein, which activates glial cells and inflammatory cytokines. (**i**) Hypomethylation of the intron 1 of *SNCA* locus dysregulates *SNCA* gene transcription, leading to abnormal α-synuclein aggregation in the cytoplasm. This allows abnormal α-synuclein to enter the nucleus following oxidative stress and sequester DNMT1 into the cytoplasm, enhancing the hypomethylation of *SNCA* gene and causing dysregulated transcription. (**ii**) Meanwhile, HDAC inhibitors cause hyperacetylation of α-synuclein linked histones H2, H3, H4, and p300 promoter region of *SNCA* to induce α-synuclein aggregation. (**iii**) Synergic interaction of DNMT1, circSNCA, and cirs-7a suppresses miR-7 expression, which upregulated *SNCA* gene expression to trigger α-synuclein accumulation. (**B**) Similarly, LncHOTAIR1 and lncMALAT1 interact with miR-205 to overexpress *LRRK2* and activates MAPK signaling pathway. (**C**) miR-34b/c interacts with *PARK2* and *DJ-1* to inhibit their neuroprotective role and activates glia cells. miR-494 and miR-4639 downregulate *DJ-1* activity and upregulate nitric oxide ten folds to promote neuroinflammation. Moreover, lower expression of *PGC-1α* also contributes to MAPK signaling pathway activation. Altogether, these epigenetic mechanisms interact with each other and with MAPK signaling pathways to induce neuroinflammation in PD development. Note: ↓ shows downregulated expression, and ↑ shows an upregulated expression.

Furthermore, various epigenetic modifications also induce α-synuclein accumulation, which triggers dysfunctional MAPK signaling pathway induced neuroinflammation (Figure 3). For instance, circSNCA and cirs-7 have been observed to inhibit the neuroprotective function of miR-7, which causes upregulated expression of α-synuclein and results in consequent neuroinflammation in PD models (Figure 3A (iii)) [70]. Moreover, lower methylation of *SNCA* also causes higher translation of *SNCA* mRNA and α-synuclein aggregation [93,94,95]. Abnormal α-synuclein aggregation further prevents the translocation of DNMT1 enzyme into the nucleus to dysfunctional its activity and exacerbate hypomethylation of upstream *SNCA* gene (Figure 3A (i)) and other related genes in PD affected models [166]. However, further investigation shows that increased levels of α-synuclein directly bind to histones that overexpress the histone acetyltransferase and lower the acetylated histone H3 (Figure 3A (ii)) [140]. This process led to reduced sirtuin activity and increased α-synuclein aggregation along with mitochondrial dysfunction and oxidative stress, resulting in DA damage and neuroinflammation [141]. In a nutshell, we conclude that DNMT1, circSNCA, and cirs-7a work synergistically to induce α-synuclein accumulation and disrupt the normal functioning of the MAPK signaling pathway, aggravates the neuroinflammation (Figure 3A). However, exploration of the precise mechanism needs further research.

Similarly, *LRRK2* is another important PD-associated gene, containing various functional domains such as leucine-rich repeats, a Ras-related GTPase domain, a MAP3K domain, and WD-40 repeats [167]. It is reported that the abnormal function of the Ras-related GTPase domain and a MAP3K domain of *LRRK2* are strongly associated with the dysfunctional MAPK signaling pathway and lead to mitochondrial dysfunction, oxidative stress, and neuronal apoptosis [168,169,170]. In the past few years, various epigenetic mechanisms have been found that dysregulate the *LRRK2* gene in PD studies. In MPP^+^-induced PD mouse model, Liu et al., revealed that LncRNA MALAT1 (metastasis-associated lung adenocarcinoma transcript 1) downregulates the miR-205-5p and activates the immune response and neuroinflammation [81]. Similarly, in the same year, lncRNA HOTAIR (HOX Transcript Antisense Intergenic RNA) was also found to target miR-205-5p and shown similar neurotoxic effects in the PD model [80]. Therefore, it is predicted that LncRNA MALAT1 and lncRNA HOTAIR interact synergistically to downregulate the miR-205-5p and influence the neuroinflammation in parkinsonism (Figure 3B).

Moreover, under normal circumstances, the *Parkin* gene displays neuroprotective properties by inhibiting the JNK signaling pathway [171]. However, in PD, the *Parkin* gene is dysregulated that causes mitochondrial dysfunction and ROS production that mediated neuronal inflammation. It is presumed that *Parkin* dysregulation activates the JNK signaling pathway that contributes to the pathological characterization of PD. Besides hereditary mutations, overexpression of miR-34b/c is found to reduce *Parkin* and *DJ-1* gene expression in PD models, subjugating that miR-34b/c activates JNK signaling pathway to feed-forward parkinsonism [172,173]. Similarly, *DJ-1* also serves as a neuroprotective protein and protects neurons against oxidative damage by inhibiting the JNK signaling pathway by targeting the *MEKK1* [174]. Interestingly, it also mediates inflammatory responses in astrocytes and α-synuclein accumulation in neurons [175]. However, *DJ-1* deficiency causes p38 signaling pathway activation in astrocytes that upregulates inducible NO synthase (iNOS) to produce 10-fold more NO (nitric oxide) and aggravates the neuroinflammation [175]. An investigation of PD patients’ plasma samples shows that upregulated miR-4639-5p triggers oxidative stress-induced lesions by directly targeting the *DJ-1* gene [176]. Likewise, a similar expression of miR-494 in the substantia nigra of post mortem PD patients downregulates the *DJ-1* expression and leads to subsequent neuronal inflammation (Figure 3C) [177]. Conclusively, these studies affirm that epigenetic modifications play a dominant role in regulating MAPK signaling pathways for progressive neuroinflammation in PD.

### 4.2. PI3/Akt/mTOR Pathway in Neuroinflammation

The P13K/Akt/mTOR signaling pathway is a universally expressed intracellular signaling pathway and controls several key processes, including inflammatory responses, cellular activation, and cell death [159]. This pathway contains a trilogy of lipid kinases, namely P13K (phosphoinositide 3-kinase), Akt (As protein kinase B), and mTOR (mammalian target of rapamycin), that works in coordination to trigger cascades of reactions, resulting in the NF-κB translocation. P13K activates Akt, which further triggers mTOR. It is speculated that activation of microglia causes the activation of the P13K/Akt/mTOR signaling pathway, which intensifies NF-κB activity and releases inflammatory molecules such as iNOS COX-2 [159,178]. Numerous studies have shown that P13K/Akt/mTOR signaling pathway is highly activated in PD models, indicating its prominent role in modulating neuronal damage and associated inflammations.

**Figure 4:**
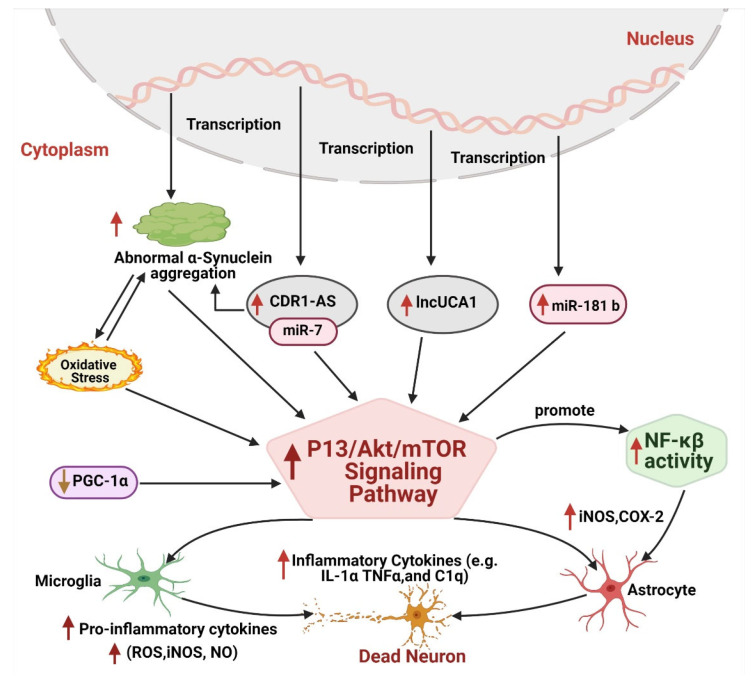
The landscape of synergic epigenetic interactions with P13K/Akt/mTOR signaling pathway in PD development. P13K/Akt/mTOR signaling pathway is activated due to overexpression of lncRNA UCA 1 and miR-181b, which leads to ROS production and other inflammatory responses. Similarly, CDR1-AS sponge with miR-7 and increases mTOR signaling. Moreover, lower expression of *PGC-1α* also regulated the inflammatory phenotype of microglia and enhanced P13K/Akt/mTOR pathway signaling. Activated P13K/Akt/mTOR signaling pathway enhances NF-κβ activity that further releases pro-inflammatory cytokines along with iNOS and COX-2. Altogether, these epigenetic mechanisms interact with each other and with P13K/Akt/mTOR signaling pathways to induce neuroinflammation in PD development. Note: ↓ shows downregulated expression, and ↑ shows an upregulated expression.

Converging evidence has shown that various epigenetic modifications directly target P13K/Akt/mTOR signaling pathways to trigger neuroinflammation and neuronal apoptosis in PD (Figure 4). For instance, upregulated expression of lncUCA directly links with activated P13K/Akt/mTOR signaling in PD studies [78], showing that lncUCA1 plays a prominent role in neuroinflammation in PD. Similarly, miR-181b family, highly regulated and expressed in astrocytes, is known for its prominent role in PD pathogenesis. Various PD studies have shown that upregulated miR-181 induces autophagy and inflammation through P13K/Akt/mTOR signaling pathways [179]. This observation was confirmed by other studies when overexpression of miR-181c in LPS cultured astrocytes released more inflammatory cytokines, resulting in neuronal death. Whereas its knockdown enhanced the production of glial cell-derived neurotrophic factor (GDNF) and vascular endothelial cell growth factor (VEGF) in astrocytes and protected against dopaminergic neurons oxidative stress. Moreover, its downregulated expression also inhibited the production of inflammatory proteins, such as glutathione peroxidase 1 and 4 (Gpx1 and Gpx4), peroxidase 2 (Prdx2), and glutaraldehyde toxin (SH3BGRL3), and reduced astrocytes inflammation [180]. In another study, lincRNA-p21 was observed to sponge with miR-181 and induced microglia activation through upregulating PKC-δ inflammation processes. lincRNA-p21 is functionally associated with p53 during cell proliferation, cell programming, and apoptosis, and all of these events depend on the competitive binding of the miR-181 family with lincRNA-p21 (Figure 4). Downregulation of miR-181 has reduced the linkage of p53/lincRNA-p21/miR-181 family/PKC-δ and presented therapeutic effects by reducing microglia activation through the downregulation of iNOS, NO and ROS, and the pro-inflammatory activity of lincRNA-p21 [181]. Further, it was found that DNA methylases (DNMT) also regulate the expression of miR-181 in astrocytes. Ji et al., (2017) investigated that PPARβ/δ (peroxisome proliferator-activated receptors) displays a neuroprotective role by reducing the expression of DNMTs. This causes hypermethylation of CpG islands in the *miR-181a* gene, resulting in the silencing of miR-181a and protection of astrocytes against damage induced by ER stress [182]. Together, these findings suggest that antagonizing miR-181 by inhibitor-lincRNAp21 and DNMT may hinder the neurotoxicity of P13K/Akt/mTOR signaling pathways and reduce the activate microglia and neuroinflammation in PD pathogenesis.

Intriguingly, CDR1-AS, as the most abundant circRNA, has been widely associated with neurological disorders. CDR1-AS contains more than 70 sites for miR-7 and sponge effectively with miR-7. It is normally involved in the storage and transportation of miR-7. A body of evidence has shown that the CDR1-AS-miR-70 axis plays a significant role in neuroinflammation through the upregulating mTOR signaling pathway and contributes to PD pathogenesis (Figure 4) [183]. Moreover, CDR1-AS also sponges with miR-671 and mediates CDR1-AS Ago2 induced degradation that works as a protective mechanism against neuronal damage. During stressful conditions, miR-671 binds with the CDR1-AS-miR-7 axis and degrades CDR1-AS. This process releases miR-7, which provides neuroprotection by downregulating the mTOR signaling pathway and lowering α-synuclein aggregation to combat neuronal apoptosis (Figure 4) [184]. Thus, CDR1-AS-miR-70/miR-671 complex can serve as an effective benchmark therapy to treat PD patients in the future.

### 4.3. JAK/STAT Signaling Pathway

**Figure 5:**
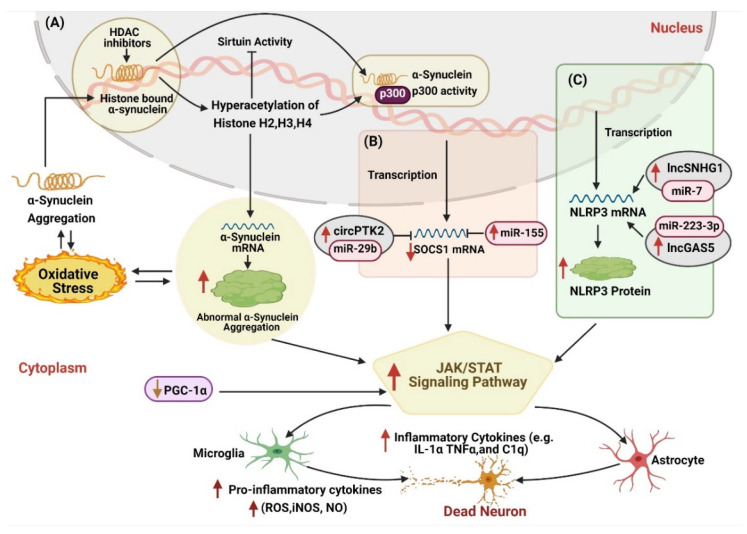
The landscape of synergic epigenetic interactions with JAK/STAT signaling pathway in PD development. JAK/STAT signaling pathway activation involves multiple epigenetic events. (**A**) Hyperacetylation of Histone H2, H3, and H4 results in the overexpression of the *SNCA* gene, which leads to α-synuclein aggregation and JAK/STAT signaling pathway activation. (**B**) Higher expression of miR-155 and circPTK2 sponging with miR-29b inhibit *SOCS1* expression that accelerates neuronal apoptosis through SOCS-1-JAK2/STAT3-IL-1β signaling. (**C**) Similarly, synergic interaction of lncSNHG1 sponging with miR-7 and lncGAS5 sponging with miR-223-3p activates NLRP3 inflammasome, which transforms glial cells to the inflammatory phenotype and the aggregation of α-synuclein. Moreover, lower expression of *PGC-1α* also contributes to JAK/STAT signaling pathway activation. Altogether, these epigenetic mechanisms interact with each other and with JAK/STAT signaling pathways to induce neuroinflammation in PD development. Note: ↓ shows downregulated expression, and ↑ shows an upregulated expression.

The Janus kinase/signal transducers and activators of transcription (JAK/STAT) signaling pathway are known to regulate several key cellular processes, including cell proliferation, differentiation, apoptosis, and immune regulation [185]. Usually, cytokines (IFN-g) employs JAK/STAT signaling pathway as their principal mode of signaling. It consists of four cytoplasmic tyrosine kinases: JAK1, JAK2, JAK3, and TYK2, triggering tyrosine phosphorylation upon cytokine receptor binding [186,187]. JAKs further operates seven latent transcription factors named STATs; STAT1-4, STAT5a, STAT5b, STAT6) through tyrosine phosphorylation [186,188,189,190]. Under stressful conditions, STATs phosphorylate and translocate from the cytoplasm into the nucleus. They target specific cis-elements such as GAS or gamma activated sequence and ISRE or interferon-stimulated regulating element to regulate various genes [188]. Dysregulation of the JAK/STAT pathway represents the pathogenic phenotypes of various neurogenerative and neuroinflammatory diseases [191,192]. A study on the PD model showed that overexpression of α-SYN leads to the activation of the JAK/STAT pathway (Figure 5A) [193]. However, inhibition of the JAK/STAT pathway lowers the microglia proliferation and macrophage infiltration and reduces the MHC class II expression [194]. Thus, the JAK/ STAT signaling pathway induces innate and adaptive immune responses in Parkinson’s disease. However, JAK inhibitor might provide viable insight towards future therapeutics for PD patients.

A large number of studies have shown that stimulation of mouse microglia with aggregated α-syn led to the expression of MHC class II and the production of NO, TNF-α, and IL-1β, which induced chronic inflammation in PD [193,195,196,197]. IFN-γ and IL-6 are the two most effective activators of the JAK/STAT pathway found elevated in PD [198,199,200]. IFN-γ regulated the activity of microglia through the JAK/STAT pathway and participated in the death of dopaminergic neurons in the PD model. Moreover, it also promotes the polarization of macrophages through JAK/STAT pathway [198]. A growing body of evidence has shown that epigenetic mechanism contributes effectively to modulating JAK/STAT pathway (Figure 5). An investigation on the PD model showed that upregulated miR-155 overexpresses *SOCS1* gene expression, which triggers JAK/STAT pathway [46]. Another study performed by Wang et al., 2018 demonstrated that circPTK2 sponges with miR-29b and activate SOCS-1-JAK2/STAT3-IL-1β signal transduction which causes microglia activation and inflammatory factor secretion, leading to neuronal apoptosis [201]. In another study, downregulation of DNMT1 in nestatin-positive NPCs enhanced GFAP and S100 expression and resulted in JAK/STAT pathway, including *STAT1* and *STAT3* [202]. Therefore, we speculate that the DNMT1 enzyme and circPTK2-miR-29b may act on different target genes to synergistically activate the JAK/STAT pathway, which brings about the activation of microglia and other inflammasomes and promote the production of inflammatory factors (Figure 5B).

Oxygen glucose deprivation (OGD) is one of the influential factors that trigger the release of pro-inflammatory cytokines and induces neuronal damage to accelerate neuroinflammation [203]. Recently, a study on the PD mouse model showed that knocked out of the *PINK1* gene resulted in OGD-induced ischemic damage, which leads to neuronal damage through mitochondrial dysfunction and oxidative stress [204]. An investigation on OGD-activated microglia reported that circPTK2 sponge with miR-29b to downregulate its expression. This interaction reduces *SOCS-1* gene expression and activates the JAK2/STAT3 signaling pathway, which in turn increases the production of TNF and IL-1β, induced microglial neuronal damage (Figure 5B) [201]. However, upregulation of miR-29b protected damaging neurons against OGD by suppressing the JAK2/STAT3 signaling pathway [201]. Thus, silencing of circPTK2 or upregulation of miR-29b may present therapeutic potential against diseases related to OGD-induced microglial activation, especially PD.

Additionally, the NLRP3 inflammasome is found to interact with various components of the JAK/STAT pathway, implicating their role in PD pathogenesis. [205,206]. Activation of NLRP3 induced the activation of the caspase-1 enzyme and triggered the production of pro-inflammatory cytokines such as IL-1β and IL-18 [207]. Convincing studies have shown that lower expression of miR-7 is associated with the accumulation of α-syn, the loss of dopaminergic cells, and the decrease of dopamine in the striatum, hallmark PD [208]. lncSNHG1 was observed as a competitive endogenous RNA of miR-7 that regulates the expression of *NLRP3* gene and causes the activation of the NLRP3 inflammasome (Figure 5C). In the microglial supernatant transfer model, knockdown of lncSNHG1 or *NLRP3* in BV2 cells stimulated by LPS inhibited the apoptosis of primary neurons and the increase of caspase-3 activity. Whereas, the downregulation of lncSNHG1 increased the expression of miR-7 and inhibited the activation of microglia and NLRP3 inflammasomes in the substantial nigra compact part and the loss of dopaminergic neurons of the MPTP mice [209]. These results indicate that lncSNHG1 promotes neuroinflammation in the pathogenesis of PD by regulating the miR-7/NLRP3 pathway (Figure 5C).

Further studies have shown that miR-223-3p is a negative regulator of NLRP3 protein (a key protein of inflammasome) involved in regulating pathological processes in various disorders, including cancer, autoimmune and inflammatory diseases, etc. Roberta Mancuso et al., in 2019, reported that the serum concentration of miR-223-3p could be a differential diagnosis and serve as a potential non-invasive biomarker for AD, PD, and mild cognitive impairment (MCI) [210]. Another investigation has shown that lncGAS5 was found upregulated in the LPS-treated PD mouse model. LncGAS5 sponge miR-223-3p to upregulate the expression of GAS5. GAS5 promotes microglial-induced inflammation by regulating *NLRP3*, which accelerated neuroinflammation in PD (Figure 5C) [211]. Taken together, these various findings suggest that JAK/STAT signaling pathways participates in neuroinflammatory responses and neuronal apoptosis, triggered by dysregulated epigenetic modifications in the development of PD.

### 4.4. NF-κβ Activation in Neuroinflammation Pathway

The NF-κB family is highly expressed in the brain, especially in neurons, glial cells, and Schwann cells, mostly as p50/p50 homodimers and p50/RelA heterodimers [212]. It plays a significant role in modulating inflammation and apoptosis and regulates the brain programming of systemic aging and the pathogenesis of several brain disorders [213,214]. Accumulating evidence has shown phenomenal changes and deregulation of NF-κβ in Parkinsonism. Post-mortem reports of various PD patients revealed overexpressed RelA nuclear translocation in melanized neurons in SNpc, highlighting the activation of the NF-κβ signaling pathway in PD [215]. Several other studies show that the NF-κβ pathway contributes to α-syn-deposition associated neuronal damage [216,217]. Other investigations supported these findings showing that α-synuclein internalization in microglia influences the nuclear aggregation of RelA and induces microglia activation. [218]. Recently, Wang et al., (2020) corroborated lower levels of cRel in the SNcp and striatum of the PD mouse model and blood samples of PD patients. Moreover, cRel was found to promote the overexpression of apoptotic genes to promote PD-related neuronal degradation [219], thereby supporting the idea that dysregulated NF-κβ pathway induces microglia activation.

**Figure 6:**
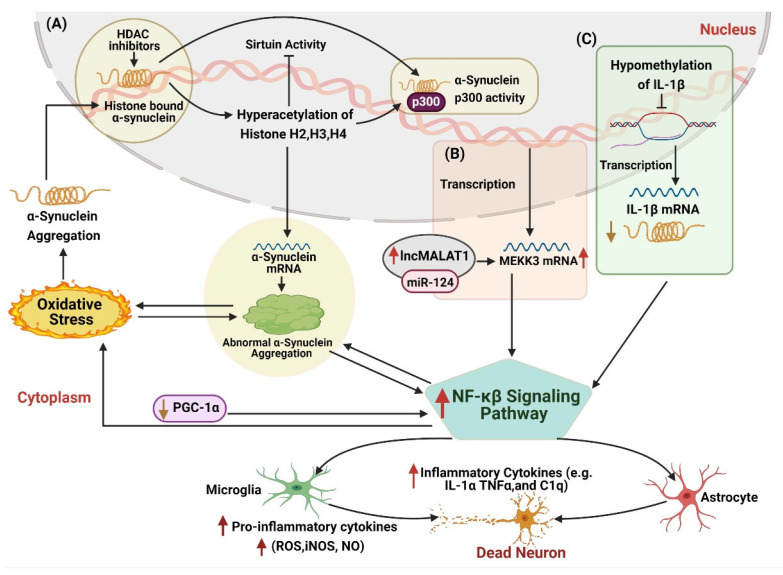
The landscape of synergic epigenetic interactions with NF-κB signaling pathway in PD development. The NF-κB signaling pathway is activated on the higher level of α-synuclein deposits. (**A**) Increased histone H3 acetylation overexpresses the *SNCA* gene and increases the release of inflammatory cytokines. (**B**) Similarly, coordination of lncMALAT1 sponging with miR-124 and *MEKK3* also enhances NF-κB signaling pathway activation. (**C**) Hypomethylation of IL-β displayed similar neuroinflammatory effects. Moreover, lower expression of *PGC-1α* also contributes to NF-κB signaling pathway activation. Altogether, these epigenetic mechanisms interact with each other and with NF-κB signaling pathways to induce neuroinflammation in PD development. Note: ↓ shows downregulated expression, and ↑ shows an upregulated expression.

Various epigenetic modifications play an active part in modulating the NF-κβ pathway to induce neuroinflammation and neurodegeneration (Figure 6). Activated NF-κB signaling in the rat MPTP model showed higher histone H3 acetylation in the *SNCA* promoter region, leading to α-syn accumulation (Figure 6A). However, inhibition of NF-κβ signaling reduced H3 acetylation of the *SNCA* gene and mediated therapeutic effects against motor dysfunction [220]. Hypomethylation of IL-1β also contributed to the activation of the NF-κβ pathway, triggering neuronal damage and neuroinflammation (Figure 6C) [79,100]. Consequently, miR-124 is one of the highly expressed miRNAs in the brain, involved in neurotransmission, neuroinflammation, neurogenesis, autophagy, mitochondrial function, etc. It also participates in the maintenance of the microglia resting stage. However, its downregulated expression brings about microglial activation [221]. Previous literature has shown that miR-124 contributes to PD pathogenesis by modulating cell survival, cell damage, oxidative stress, and neuroinflammation through calpain 1/p25/cyclin-dependent kinases 5 (CDK5), nuclear factor kappa B (NF-κβ), signal transducer, and activator of transcription 3 (STAT3), BCL-2-interacting mediator of cell death (Bim) and extracellular signal-regulated kinase (ERK) pathways [222,223,224,225]. This finding was further corroborated by other PD studies, where lower expression of miR-124 reduces the expression of anti-apoptotic gene *BIM* and causes the overexpression of apoptotic proteins BCL-2, resulting in neuronal apoptosis [223,224,225].

Additionally, lncMALAT1 is found to be closely related to miR-124. It regulated breast cancer progression endogenously by downregulating miR-124 and activating the CDK4/E2F1 signaling pathway [226]. Moreover, lncMALAT1 competed with miR-124 to regulate the GRB2 expression and promoted the growth and invasion of HR-HPV cells [227]. A study on the PD model revealed that lncMALAT1 promoted DA neuron apoptosis through the NF-κβ signaling pathway by sponging with miR-124 [50], thereby suggesting that the lncMALAT1-miR-124-MEKK3/NF-κβ axis may provide new insight for the treatment of the neuroinflammation caused by the activation of microglia (Figure 6B).

Interestingly, overexpression of miR-124 displayed neuroprotective effects on damaging neurons against neuroinflammation. In 2013, Sun et al., showed that upregulated miR-124 performs anti-inflammatory action by inhibiting the production of pro-inflammatory cytokines [228]. Further studies showed that *MEKK3*, as a direct target of miR-124, inhibited neuroinflammation by regulating the MEKK3/NF-κβ signaling pathway. Further, it reversed the expression of LPS-induced pro-inflammatory cytokines and promoted the secretion of neuroprotective factors [69]. To sum up, dysregulated miR-124 plays a dual role in neuroinflammation and may serve as an effective biomolecule for devising PD management strategies.

## 5. Conclusion and Perspectives

Since the 19th century, when James Parkinson coined the name Parkinson’s disease, researchers have never stopped exploring its etiology and pathogenesis. A significant number of studies have proposed a strong association between inflammatory response, oxidative stress, and PD. During PD progression, neuroinflammation plays a vital role in the initiation and progression of PD. It is observed that the central nervous system’s inflammation activates glial cells and cytokine release, whose abnormal function leads to progressive degeneration of dopaminergic neurons in the substantia nigra and striatum. This pathological activity enhances the neuroinflammatory process by forming a vicious circle and promotes advanced PD development, thus hinting at the involvement of key cellular and molecular events in PD that are barely investigated thoroughly and make PD treatment more challenging. However, the hidden mystery of these mechanisms underlying the glial cells mediated neuroinflammation and neurotoxicity in PD is an active area of investigation that may provide a new avenue for better understanding of the PD pathology and may help in the establishment of target-specific PD drugs.

**Figure 7:**
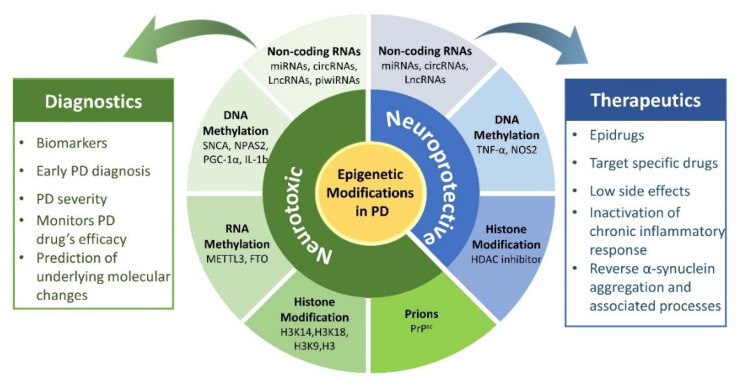
Proposed applications of neurotoxic and neuroprotective epigenetic modification in Parkinson’s disease management. Neurotoxic epigenetic modifications can be utilized for PD diagnosis. In contrast, neuroprotective epigenetic modifications can be utilized for PD therapeutics.

Recently, epigenetics has garnered increased interest because of its significant contribution to the central nervous system’s development. Notably, various epigenetic mechanisms regulate inflammatory responses, activation of glial cells, and inflammatory cytokines. To investigate their role in the onset and progression of neuroinflammation in PD pathogenesis, we thoroughly reviewed these epigenetic mechanisms. These epigenetic mechanisms act as a dual-edge sword, presenting neurotoxic and neuroprotective characteristics. Neurotoxic epigenetic modifications work synergistically to promote chronic neuroinflammation in PD models. For instance, miR-7 and lncSNHG1, miR-223-3p, and lncGAS5 interact together to promote the pathogenesis of PD inflammation through *NLRP3* activation and can be utilized in future early PD diagnosis. However, neuroprotective epigenetic modifications protect DA neurons against oxidative stress and neuroinflammation. Antagonism of CDR1AS and circSNCA with miR-7 has been observed to prevent DA neuron damage and associated neuroinflammation; thus, it has been proposed for future PD therapy. Hence, dual characteristics of these epigenetic modifications make them ideal candidates for future PD management, as illustrated in Figure 7. Further, we have observed that these epigenetic modifications are interlinked and work in coordination to target neuroinflammatory pathways specifically to trigger PD progression. Methylation of miR-155, miR-124-3p, lncUCA1, and TNF-α promoter regulate neuroinflammation through the JAK/STAT pathway. Similarly, hypomethylation of miR-124 and il-1β activates the NF-ΚB pathway to regulate neuroinflammation. Henceforth, we believe that the synergy between epigenetic mechanisms and neuroinflammatory pathways in PD results in the activation of microglia and astrocytes, which leads to pyrolysis and aggravates neuronal oxidative stress, thus inducing the aggregation of α-synuclein and other proteins. However, further study in this domain may reveal new dimensions in PD research and provide novel insights for improved diagnosis and treatment of Parkinson’s patients.

## Figures and Tables

**Table 1:** MicroRNAs involved in the neuroinflammation in Parkinson’s disease. ↑ shows up-regulated, ↓ shows down-regulated.

microRNAs	Model	Target	miRNA Expression	Function	References
**miRNA with Neuroprotective Effect**					
**miR-7**	PD patients, MPTP mice model, MPP^+^-SH-SY5Y cell model	*SNCA*	↑	Downregulate α-synuclein and acted as a neuroprotective agent against oxidative stress, mitochondrial impairment, and neuroinflammation	[47]
**miR-7**	MPTP mouse model	*NLRP3*	↑	Downregulate NLRP3 expression and reduced neuronal damage, and improved microglia function	[48]
**miR-182-5p** **miR-183-5p**	Primary neuronal cell model of midbrain	*GDNF*	↓	Mimic glial cell-derived neurotrophic factor (GDNF) and protected the survival of DA neurons	[49]
**miR-124-3p**	MPP^+^-SH-SY5Y cell model	*STAT3*	↓	Downregulate the production of caspase-3, inflammatory factors TNF-α, IL-1β, and reactive oxygen species	[50]
**miR-124**	LPS-BV-2 cell models, MPTP mice model, MPP^+^-SH-SY5Y cell model	*MEKK3*	↓	Downregulate the activation of microglia, the production of pro-inflammatory factors, and cell apoptosis	[51]
**miR-195**	LPS-BV-2 cell model	*ROCK1*	↓	Downregulate the release of pro-inflammatory cytokines (iNOS, IL-6, and TNF-α) while upregulating the release of anti-inflammatory cytokines (IL-4 and IL-10)	[52]
**miR-190**	LPS-BV-2 cell model, MPTP mice model	*Nlrp3*	↓	Downregulate pro-inflammatory cytokines and microglia activation	[53]
**miR-146a**	Human glial cell lines	*IL-6 and COX-2*	↑	Inhibit inflammatory response mediated by glial cells and provide neuroprotection.	[54]
**miRNA with Neurotoxic Effect**					
**miR-155**	Microglia and astrocytes cultured from DJ-1-knockout mouse brain	*SOCS-1*	↑	Upregulate the expression of pro-inflammatory mediators in microglia and astrocytes	[46]
**miR-133b**	Pitx3 mutant Aphakia mice, 6OHDA-treated mice	*Pitx3*	↓	Downregulate Pitx3 and induced DA neuronal damage and trigger neuroinflammation	[40]
**Let-7**	Animal, human biopsies, cell lines, and primary cell cultures	*TLRs*	↑	Let-7 act as a signal activator of TLR7 in microglia and induced an inflammatory response in PD	[41,42,43]

**Table 2:** CircRNAs involved in the neuroinflammation in PD. ↑ shows up-regulated, ↓ shows down-regulated.

Circular RNA	miRNA Sponge	Expression	Target	Study Model	Function	References
**circSNCA**	miR-7	↓	*SNCA*	MPTP-mouse model MPP^+^-SH-SY5Y	Upregulate miR-7 that downregulate *SNCA* and reduce cell apoptosis, and improves autophagy	[61]
**mmu-circRNA-0003292**	miRNA-132	↑	*NR4A2*	MPTP- mouse model	Downregulate *NR4A2* to impair DA neurons and promote neurodegeneration and neuroinflammation	[62]
**circDLGAP4**	miR-134-5p	↓	*CREB*	MPP^+^-SH-SY5Y cells, MPTP mouse models	Regulate the activation of the CREB signal to affect the expression of *BDNF*, *Bcl-2,* and *PGC-1α* in cells, and exert neuroprotective effects.	[63]
**circHIPK2**	miR124-2HG	↑	*SIGMAR1*	Sigmar1KO mice, human astrocytoma cell line A172	Regulates astrocyte activation, autophagy, and endoplasmic reticulum stress	[64]
**circzip-2**	miR-60	↑	*Zip-2*	Transgenic C. elegans model of PD	Target miR-60 to protect DA neurons	[65]
**circSLC8A1**	miR-128	↑	*Ago2*	Substantia nigra of PD patients and healthy donors	Promote oxidative stress	[66,67,68]
**mmu_circRNA_0001320**	miRNA-124	↑	*MEKK3/NF-κB signaling pathway*	LPS-treated BV2 cells and MPTP-mouse model	Sponge with miR-124 to induce neuroinflammation through MEKK3/NF-*κ*β signaling pathway	[62,69]

**Table 3:** Long non-coding RNAs involved in neuroinflammation in Parkinson’s disease. ↑ shows up-regulated, ↓ shows down-regulated.

LncRNAs	Study Model	LncRNA Expression	Target	Function	References
**LncRNA UCA1**	6-OHDA rat model	↑	PI3K/AKT	Upregulated oxidative stress and inflammation through PI3K/Akt signaling pathway	[78]
**LncRNA HOTAIR**	MPP^+^-SH-SY5Y cells, MPTP-mouse models	↑	miR-126-5p	Regulate miR-126-5p and RAB3IP to promote the progression of PD.	[79]
**LncRNA-HOTAIR**	MPP^+^-SH-SY5Y cell model, MPTP-mice model	↑	miR-205-5p-*LRRK2*	Upregulated *LRRK2* expression by miR-205 inhibition and induce neuronal apoptosis and neuroinflammation	[80]
**LncRNA-MALAT1**	MPP^+^-SH-SY5Y cell model, MPTP-mice model	↑	miR-205-5p-*LRRK2*	Upregulated *LRRK2* expression by miR-205 inhibition and induce neuronal apoptosis and neuroinflammation	[81]
**LncRNA-AS Uch1**	iMN9D cells, MPTP-mice model	↓	*Nurr1*	Downregulated ASUch1 expression is regulated by downregulated *Nurr1* activity, results in dopaminergic dysfunction	[82]
**Lnc-p21**	MPP^+^-SH-SY5Y cells	↑	miR-625	Target miR-625 to regulate *TRPM2* expression that increase the levels of ROS, TNF-α, IL-1β and IL-6, and trigger cell apoptosis	[83]
**LncRNA AL049437**	MPP^+^-SH-SY5Y cells, MPTP-mouse model	↑	miR-205-5p	Regulated the miR-205-5p/MAPK1 axis to increase the levels of inflammatory factors and ROS	[84]
**LncRNA NEAT1**	MPP^+^-SH-SY5Y cells, MPTP-mouse model	↑	*PINK1*	Inhibited the degradation of *PINK1* to increase autophagy and displayed a neuroprotective role in PD	[85]

**Table 4:** DNA methylation involved in the neuroinflammation in Parkinson’s disease. ↑ shows up-regulated and ↓ shows down-regulated.

DNA Methylation	Model	Target	Expression	Function	References
**SNCA promoter**	PD patients, healthy controls	*SNCA*	↓	*Induces* α-syn aggregation and induces DA neuronal damage and activates glial cells to trigger neuroinflammation	[93,94,95]
**NPAS2 promoter**	PD patients, healthy controls	*NPAS2*	↓	Disturb the circadian rhythm of PD patients	[99]
**IL-1β promoter**	Mouse model	*IL-1β*	↓	Transform microglia to the M1 phenotype, that triggered neuronal damage and neuroinflammation	[79,100]
**PGC-1α promoter**	Human brains of PD patients and healthy controls, DM SYN mice	*PGC-1α*	↑	Increase inflammatory gene expression and ROS production	[101]
**TNF-α promoter**	PD patients, healthy controls	*TNF-α*	↓	Regulates the inflammatory phenotype of microglia	[102]
**NOS2 promoter**	PD patients, healthy controls	*NOS2*	↓	Reduce NO production to avoid activation of microglia	[103]

## Data Availability

Not applicable.

## Abbreviations

PD	Parkinson’s disease
AD	Alzheimer disease
LBs	Lewy bodies
CNS	central nervous system
SN	substantial nigra
SNpc	substantia nigra par compacta
DA	dopaminergic neurons
ncRNA	non-coding RNA
TNF	tumor necrosis factor
ROS	reactive oxygen species
NOS	nitric oxide synthase
NO	nitric oxide
TGF	transforming growth factor
IGF-1	insulin-like growth factor-1
LPS	lipopolysaccharide
TLR7	toll-like receptors 7
GMF	glial maturation factor
3’UTR	3’untranslated region
Act1	NF-kB activator 1
GMF	glial maturation factor
PPX	pramipexole
H3K27	methylation of histone 3 lysine 27
Jmjd3	jumonji-domain Protein 3
GDNF	glial cell-derived neurotrophic factor
MAPK	mitogen-activated protein kinase
PPX	pramipexole
miRNA	microRNA
circRNA	circular RNA
LncRNA	long coding RNA
lncRNA UCA-1	urothelial carcinoma-associated-1
LncRNA-p21	long non-coding RNA-p21
lncRNA NEAT	nuclear-enriched assembly transcript-1
LncRNA MALAT1	metastasis-associated lung adenocarcinoma transcript 1
lncRNA HOTAIR	HOX Transcript Antisense Intergenic RNA
LncRNA AS Uchl1	antisense to the mouse Ubiquitin carboxy-terminal hydrolase 1
piRNAs	Piwi interacting RNAs
piRNAs	Piwi interacting RNAs
SINEs	short interspersed nuclear element
LINEs	long interspersed nuclear element
DNMT	DNA methyltransferase
IL-1	Interleukin 1
m6A	RNA methylation
FTO	obesity related protein
ALKBH5	alkylated DNA repair protein alkB homolog 5
NMDA	N-methyl-D-aspartate
METTL3	Methyltransferase-like 3
HDAC	histone deacetylase
HAT	histone acetyltransferase
H3K14	histone H3 lysine 14
H3K18	histone H3 lysine 18
H3K9	histone H3 lysine 9
SB	sodium butyrate
TSA	trichostatin A
SAPK	stress-activated protein kinases
JNK	Jun amino-terminal kinases
ICAM-1	intercellular adhesion molecule-1
NO	nitric oxide
P13K	phosphoinositide 3-kinase
Akt	As protein kinase B
mTOR	mammalian target of rapamycin
GDNF	glial cell-derived neurotrophic factor
VEGF	vascular endothelial cell growth factor
PPARβ/δ	peroxisome proliferator-activated receptors
JAK/STAT	Janus kinase/signal transducers and activators of transcription
MCI	Mild cognitive impairment
CDK5	calpain 1/p25/cyclin-dependent kinases 5
NF-κB	Nuclear factor kappa B
STAT3	Signal transducer and activator of transcription 3
